# The microbiota-tryptophan-brain axis in neurodegenerative diseases: pathogenic mechanisms, disease-specific roles, and translational therapeutics

**DOI:** 10.3389/fmicb.2026.1820111

**Published:** 2026-05-20

**Authors:** Ziyi Wang, Lu Li, Yuhan Dong, Yamin Zhang

**Affiliations:** 1 The First Clinical Medical College of Gansu University of Chinese Medicine (Gansu Provincial Hospital), Lanzhou, Gansu, China; 2 Department of Neurology, Gansu Provincial Hospital, Lanzhou, Gansu, China

**Keywords:** Alzheimer’s disease, Aryl hydrocarbon receptor, Microbiota-gut-brain axis, neurodegenerative diseases, Parkinson’s disease, tryptophan

## Abstract

The pathogenesis of neurodegenerative diseases (NDDs) such as Alzheimer’s disease (AD) and Parkinson’s disease (PD) is very complex. Recent studies have shown that gut microbiota and their metabolites play a key role in the progression of these diseases. Tryptophan (Trp) is an essential amino acid, which mainly produces a variety of biologically active compounds in the intestine through the metabolism of indole pathway, Kynurenine pathway (KP) and serotonin pathway, including indole derivatives, Kynurenine (KYN) and serotonin (5-HT). These metabolites affect the central nervous system (CNS) through the Microbiota-gut-brain axis (MGBA) and affect CNS in a variety of mechanisms, including immune regulation, neuroprotection and maintenance of intestinal barrier function. They are involved in key pathological processes such as neuroinflammation, oxidative stress and pathological protein aggregation. This paper systematically reviews the mechanism of the role of Trp metabolites derived from gut microbiota in NDDs, and explores their specific roles in AD, PD, Amyotrophic Lateral Sclerosis (ALS) and Huntington’s disease (HD), and summarizes the potential therapeutic value of the current pathway strategy. These strategies include nutritional intervention, targeted microbiome therapy [such as probiotic and fecal microbiota transplantation (FMT)], and metabolite-derived drugs. Future research must clarify its dynamic mechanism in the human body, develop relevant biomarkers, and promote personalized prevention and treatment strategies through clinical transformation, so as to provide a new direction for early intervention and treatment of NDDs.

## 1 Introduction

Neurodegenerative diseases refer to a group of disorders that involve the gradual deterioration of neurons or myelin. This category predominantly encompasses AD, PD, HD, and ALS. Currently popular mechanism theories include neuroinflammation, oxidative stress, mitochondrial dysfunction, abnormal protein aggregation and MGBA (Ruz et al., 2020; Cheng et al., 2025; Chong and Souayah, 2025; Huang et al., 2025; Mohammad et al., 2025). Gut microbiota, also known as gut microbiota, refers to the sum of microbial communities living in the human intestine, including bacteria, fungi, viruses and other microorganisms. The function of gut microbiota on humans can be divided into three categories: beneficial bacteria (symbiotic bacteria), neutral bacteria (opportunistic pathogens) and harmful bacteria (pathogenic bacteria). Together, they play a crucial role in enhancing intestinal integrity or shaping intestinal epithelium, energy acquisition, defense against pathogens and immunoregulation (den Besten et al., 2013; Natividad and Verdu, 2013; Bäumler and Sperandio, 2016; Gensollen et al., 2016). Changes in the composition of gut microbiota will change brain-intestinal communication, thus affecting the function of CNS (Ojeda et al., 2021). MGBA forms a complex two-way communication network that connects the gut microbiota with the CNS through immune, neurological, endocrine and metabolic pathways. A large number of studies have shown that MGBA disorder plays a key role in the occurrence and development of NDDs (Chen et al., 2025).

In the MGBA-related mechanism, Trp metabolism is considered to be an important hub connecting gut microbiota and brain function regulation. Trp mainly plays a biological role in the body through three metabolic pathways, namely the KP, the serotonin pathway and the indole pathway. Among them, KP is the main pathway of Trp metabolism, which can produce a variety of neuroactive metabolites such as KYN, kynurenic acid (KYNA) and quinolinic acid (QUIN); the serotonin pathway mainly produces 5-HT, which regulates mood, sleep and nerves. It plays an important role in secretory function; while the indole pathway mainly relies on the gut microbiota to metabolize Trp to produce indole and its derivatives. These metabolites can participate in intestinal homeostasis by activating signaling pathways such as Aryl hydrocarbon receptor (AhR) and neuroimmune regulation (Zhang and Davies, 2016; Gao et al., 2020). As a key signal molecule in the MGBA, Trp metabolites can directly or indirectly regulate neuroinflammation, oxidative stress response and abnormal aggregation of pathological proteins in the brain through circulatory immunity, neuroendocrine and the vagus nerve, such as β-amyloid protein (Aβ) and alpha-synuclein (α-synuclein) (Iwaniak et al., 2024; Pathak et al., 2024; Brüge et al., 2025; Fan et al., 2025; Hoseini and Ghafari, 2025; Li G. et al., 2025; Zheng and Xu, 2025). Therefore, the abnormal Trp metabolism caused by intestinal microbial dysbiosis may create a favorable pathological environment for the occurrence and progression of NDDs by destroying the intestinal-brain signal transduction homeostasis.

Although there is more and more evidence that gut microbiota-tryptophan metabolism is related to NDDs, there are still some key gaps: (1) The disease-specific regulation mode of Trp metabolism pathways in different NDDs has not been fully clarified; (2) The molecular interaction between Trp metabolites and key pathological processes, including α-synuclein aggregation in PD and Aβ deposition in AD is not yet clear; (3) Research on the transformation of preclinical research results into clinical applications is relatively limited. This review aims to fill these gaps by integrating the latest progress in mechanism research and treatment development. In addition, this review intends to compare and analyze the metabolic changes of Trp in a variety of NDDs, including AD, PD, ALS, and HD, and further integrates relevant molecules. The relationships between key mechanisms, such as AhR signaling, and pathological features, such as Aβ deposition and α-synuclein aggregation, should be further investigated to more comprehensively reveal the common and distinct features of tryptophan metabolic imbalance across different NDDs.

## 2 The basic relationship between gut microbiota-tryptophan metabolism axis and NDDs

### 2.1 Metabolite types and generation routes

Trp, also known as β-indole alanine, is one of the nine essential amino acids required by the human body. Since the human body cannot synthesize Trp, it must be obtained through dietary sources, including food and animal products. The primary metabolic pathways of Trp in the gastrointestinal tract are as follows.

#### 2.1.1 Indole pathway

The gut microbiota can directly convert approximately 4%–6% of Trp in the gut into indole and its derivatives, which consist of: Indole-3-acetaldehyde (IAld), Indole-3-acetic acid (IAA), indole acrylic acid, Indole-3-aldehyde, Indole-3-lactic acid (ILA), and Indole-3-propionic acid (IPA). Indole exhibits concentration-dependent dual effects on inflammation: low concentrations (physiological levels) activate the AhR to suppress inflammation, while high concentrations (pathological levels) compete with other AhR ligands (e.g., IPA) to promote pro-inflammatory responses. Among them, IPA of intestinal origin is antioxidant and it plays a key role in neuroprotection (Sun et al., 2022; Xie et al., 2022; Ye et al., 2022; Fan et al., 2025; Owe-Larsson et al., 2025)

#### 2.1.2 KP

In the human body, over 95% of Trp is metabolized through the KP. The KP utilizes indoleamine 2,3-dioxygenase (IDO) and tryptophan 2,3-dioxygenase (TDO) to convert Trp into KYN. The conversion process of IDO-mediated Trp to KYN is mainly induced by inflammatory stimulation, of which interferon-γ (IFN-γ) is the key starting factor. In addition, other pro-inflammatory cytokines [such as tumor necrosis factor-α (TNF-α), interleukin-1β (IL-1β) and interleukin-6 (IL-6)], pathogen infection, chronic immune activation and cellular stress can also significantly enhance the activity of IDO (O’Connor et al., 2009). IDO is an intracellular monomeric enzyme that exists in two isoforms: IDO1 and IDO2. IDO1 is widely distributed across various organs, including the liver, kidney, spleen, and brain, where it plays an immunomodulatory role. In contrast, IDO2 exhibits higher specificity compared to IDO1 (Chang R. Q. et al., 2018; Hornyák et al., 2018; Nishi et al., 2018; Weng et al., 2018). TDO, a tetrameric hemoglobin encoded by the TDO2 gene, is highly expressed in human liver tissue, where it metabolizes approximately 95% of liver Trp (Li et al., 2007; Jernigan and Sun, 2017; Tiwari and Paramanik, 2025). KYN and its metabolites, such as 3-hydroxykynurenine and QUIN, can stimulate the production of free radicals, resulting in lipid peroxidation and oxidative stress, which in turn demonstrates neurotoxicity (Okuda et al., 1996; Yang et al., 2024).

#### 2.1.3 Serotonin pathway

In the serotonin pathway, Trp is metabolized by tryptophan hydroxylase (TPH1) to produce 5-HT in enterochromaffin cells (ECC). Both indole and KYN exhibit a variety of biological activities and can traverse the blood-brain barrier (BBB). Notably, while the 5-HT present in systemic circulation cannot penetrate the BBB, gut microbiota can influence CNS 5-HT signal conduction by modulating the levels of Trp in the brain. Approximately 90%–95% of the human body’s 5-HT is synthesized in the gastrointestinal tract, with only 5% produced in the raphe nuclei of the brainstem. This small percentage of 5-HT is closely associated with anxiety, depression, and other emotional disorders (Kwon et al., 2019; Coray and Quednow, 2022; Hung et al., 2025). Furthermore, it is important to emphasize that indole and kynurenine metabolites possess diverse biological activities and can cross the BBB, thereby directly or indirectly influencing CNS function (Yang et al., 2024).

**FIGURE 1:**
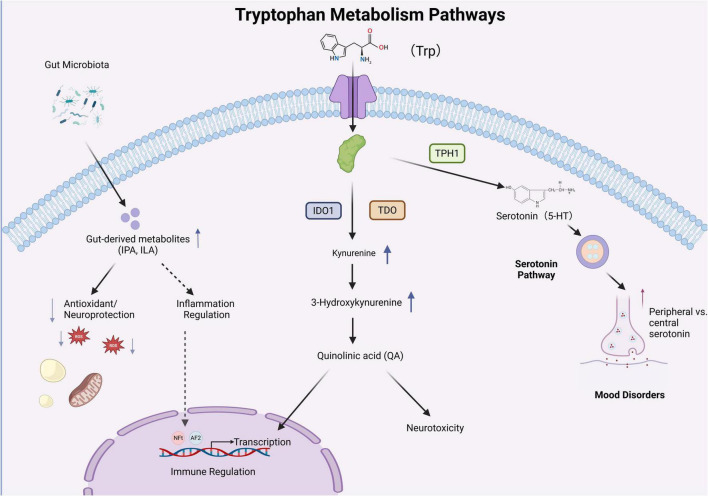
The three major metabolic pathways of tryptophan.

The gut microbiota influences the function of the CNS by modulating three primary metabolic pathways of Trp: the indole pathway, which has a neuroprotective effect; the KP, which produces a variety of metabolites, some of which may have neurotoxic effects; and the serotonin pathway, which affects neurotransmitter precursors. These metabolites can traverse the BBB, establishing a crucial communication axis between gut microbiota, metabolism, and the brain. An imbalance in this axis is closely associated with the pathogenesis of NDDs. Future investigations into the specific mechanisms of the metabolic network, along with the development of intervention strategies targeting microbiota, metabolic enzymes, and disease biomarkers, will unveil new opportunities for the prevention and treatment of NDDs (Figure 1).

### 2.2 External factors affecting Trp metabolism

External factors mainly include sex hormones, drug interventions, diet and environmental exposure, which play a key role in regulating tryptophan metabolism. Regarding gender and individual differences, Benedek et al. (2017) confirmed the potential cross-relationship between estrogen and gut microbiota, suggesting that estrogen may promote neuroprotection by influencing the composition of gut microbiota in experimental autoimmune encephalomyelitis (EAE). 17β-estradiol (E2), the most biologically active form of estrogen, has been widely shown to eliminate free radicals, enhance neuronal survival, and regulate neurotoxicity and inflammation (Khan et al., 2019; Bendis et al., 2024; Wang et al., 2025). Notably, Khan et al. (2019) demonstrated that E2 alleviates D-galactose-induced memory impairment by upregulating sirtuin 1 (SIRT1), which indirectly modulates gut microbiota composition to enhance indole production, thereby highlighting a crosstalk between estrogen signaling and the gut microbiota-Trp axis. Although direct evidence of estrogen’s ability to reduce pathological changes in AD, such as Aβ deposition or excessive phosphorylation of tau protein, remains elusive, this study provides novel insights for future investigations into hormones and NDDs. In terms of drug intervention, antibiotics have been shown to reduce gut microbiota diversity, which can directly or indirectly affect intestinal Trp metabolism. This is reflected by an increase in Trp levels in peripheral serum, a decrease in KYN concentration, and an increase in the ratios of Trp/KYN and KYNA/KYN (Desbonnet et al., 2015; Fan et al., 2025). These metabolic alterations may lead to disorders and potentially exacerbate NDDs. Jiang et al. (2017) demonstrated through a pig model of antibiotic treatment that Trp metabolism favors the KP over the serotonin pathway. Similar to sex hormones, there is currently no direct evidence that antibiotics influence the relationship between intestinal Trp metabolism and NDDs. Key evidence is still needed to establish whether these metabolic changes can directly induce or alleviate the pathological characteristics of NDDs, such as Aβ deposition. Future research must systematically elucidate how external factors, including sex hormones and drugs, drive the remodeling of the Trp metabolic pathway through alterations in gut microbiota in both animal models and clinical populations, ultimately impacting the neuropathological processes and clinical outcomes. Concurrently, targeted intervention strategies for this pathway, such as specific estrogen regulation or recovery of gut microbiota following antibiotic treatment, should be actively explored to evaluate their potential value in the prevention and treatment of NDDs.

### 2.3 Communication mechanisms of the MGBA

Microbiota-gut-brain axis is a two-way communication network that connects intestinal microbiota, gastrointestinal tract and CNS. This two-way communication mechanism not only includes the influence of intestinal microbiota on CNS function, but also includes the feedback regulation of CNS on intestinal physiological state and microbial composition. Several key components mediate these interactions, including microbial metabolites (such as tryptophan-derived metabolites and short-chain fatty acids), enteric nervous system, the vagus nerve, enteroendocrine cells, intestinal barriers, circulating immune cells, BBB and CNS-resident glial cells and neurons. Among them, tryptophan metabolites, as important signal molecules, build a bridge between intestinal microbiota and the brain by participating in immune regulation, neuroprotection and barrier maintenance of homeostasis. Together, these mechanisms lay a unified theoretical framework for understanding the role of tryptophan metabolism in MGBA (Cryan et al., 2019).

Aryl hydrocarbon receptor is a ligand-activated transcription factor, which plays an important role in immunomodulation, inflammatory signaling, epithelial barrier maintenance and cellular homeostasis (Ma et al., 2020).

**FIGURE 2:**
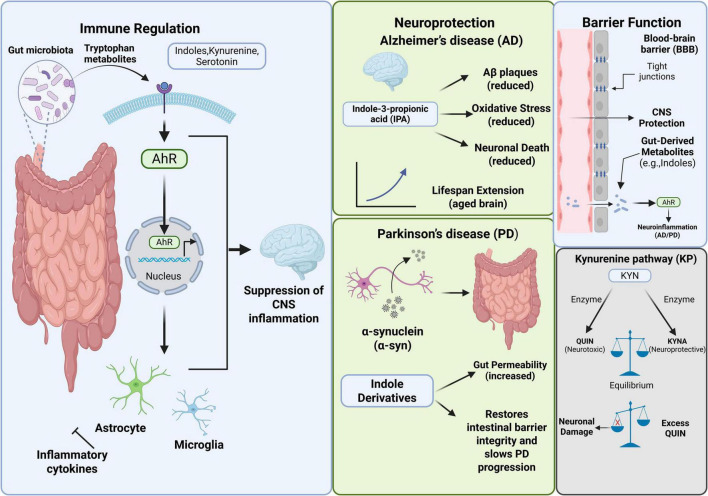
Mechanisms of tryptophan metabolites influencing the progression of neurodegenerative diseases.

The metabolism of Trp by intestinal microorganisms constitutes a key chemical basis for bidirectional communication within the MGBA. A variety of biologically active molecules produced through this process, such as indole and its derivatives, as well as metabolites of the KP, act as ligands to activate downstream signaling pathways (such as AhR), which are the key nodes to regulate brain function. These metabolites mainly affect the development of NDDs through the following three interrelated mechanisms (Figure 2).

#### 2.3.1 Immune regulation

Aryl hydrocarbon receptor serves as a transcription factor that shuttles between the cytoplasm and nucleus, and its activation influences the expression of genes associated with cellular growth, metabolism, and immune response. This receptor is integral to preserving intestinal homeostasis and is implicated in CNS disorders, including AD and epilepsy. Trp is metabolized through three pathways in the gastrointestinal tract to produce biologically active metabolites including indole and its derivatives, kynurenine pathway metabolites, and 5-hydroxytryptamine (5-HT). As the ligands of AhR, these metabolites activate AhR through the intestinal-brain immune signaling pathway. Subsequently, AhR regulates the release of inflammatory mediators by astrocytes and glial cells, and ultimately inhibits the inflammatory response in CNS (Ma et al., 2020; Salminen, 2023).

#### 2.3.2 Neuroprotection

In AD, indole metabolites such as IPA can not only reduce oxidative stress and inhibit Aβ-induced neuronal cell death, but also reduce Aβ deposition and play a neuroprotective role (Chyan et al., 1999; Sun et al., 2022). In addition, recent studies show that IPA can promote the health of the aging brain and is expected to prolong life in the future (Pappolla et al., 2021). The main pathological features of PD include degenerative changes in dopaminergic neurons in the substantia nigra, and the Lewy bodies and Lewy neurites formed by the aggregation of α-synuclein. Alpha-synuclein is a key protein to clarify the pathogenesis of PD (Bloem et al., 2021). Increased intestinal permeability may promote alpha-synuclein aggregation by activating the enteric nervous system (ENS), thus exacerbating the progression of PD (Jurcau et al., 2023). Studies show that indole and its derivatives can inhibit the increased intestinal permeability, thus slowing down the progression of PD (Sun et al., 2022; Zhou et al., 2023).

#### 2.3.3 Barrier function

Blood-brain barrier forms a selectively permeable barrier between cerebral blood vessels and brain tissue, limiting the transport of substances between blood and nerve structures. BBB plays a crucial physiological role in maintaining the integrity of brain tissue and protecting the homeostasis of CNS (Xie et al., 2019). When the integrity of BBB is impaired, exogenous harmful substances may infiltrate CNS, thus inducing NDDs (Rudzki and Maes, 2020). Metabolites (such as indole derivatives) produced by gut microbiota during Trp metabolism can pass through the damaged BBB, activate AhR, and trigger neuroinflammatory reactions. This mechanism has been confirmed in PD and AD (Salminen, 2023; Fan et al., 2025). KYN can be further metabolized into a variety of metabolites in the body, mainly including KYNA and QUIN. The existing literature generally believes that KYNA has neuroprotective or immunomodulatory properties, while the excessive accumulation of QUIN is associated with neurotoxic effects (Fan et al., 2025). However, the specific mechanism of action of these metabolites has not been fully clarified at present. Under normal circumstances, a dynamic balance is maintained between these two metabolites. However, when the production of QUIN exceeds that of KYNA, excessive QUIN can lead to neuronal death, thereby damaging the function and structure of the CNS and accelerating the progression of PD (Li et al., 2022; Fan et al., 2025).

The biologically active molecules produced by the metabolism of Trp in the gut microbiota, as the core signal transmission medium of the MGBA, jointly affect the progress of NDDs through multiple mechanisms, including immune regulation, direct neuroprotection and the function of maintaining the intestinal and BBB. Future research must further clarify the precise targets and spatiotemporal dynamics of specific metabolites in complex gut microenvironment. And give priority to exploring precise intervention strategies for AhR pathways or key metabolic balance. At the same time, the potential of these cyclic metabolites and their ratios as new biomarkers to predict disease staging and therapeutic responses should be evaluated.

## 3 The role in NDDs

### 3.1 AD

The pathogenesis of AD has not been fully clarified. At present, it is believed that it is caused by a combination of genetic factors, lifestyle and environmental factors. Mainstream theories include Aβ deposition and overphosphorylated tau protein, etc (Scheltens et al., 2021). The study of Sun et al. (2022) shows that indole is a gut microbiota metabolite that can reduce the level of Aβ deposition and tau protein phosphorylation in APP/PS1 mouse models by activating AhR and inhibiting neuroinflammation. In addition, gut microbiota disorder may contribute to reduced local Trp availability in the intestinal niche by impairing microbial Trp metabolism, which in turn has been positively associated with cognitive decline (Pan et al., 2024). It remains to be clarified whether such Trp deficiency primarily reflects diminished microbial metabolic capacity or a systemic reduction in Trp bioavailability. Moreover, indole derivatives can enhance synaptic plasticity and inhibit neuroinflammation, providing a new way to treat AD. The plasma levels of tryptophan-derived metabolites, such as IPA, which are primarily generated by gut microbial metabolism, may serve as potential biomarkers for AD. A reduction in these metabolite levels has been associated with the aggravation of cognitive impairment. Notably, while these metabolites detected in the peripheral circulation originate predominantly from microbial activity in the gut, their plasma concentrations reflect the integrated outcome of microbial production, host absorption, and systemic metabolism. At the same time, a decrease in 5-HT levels increases the risk of cognitive impairment (Wu et al., 2021; Owe-Larsson et al., 2025). It is worth noting that the Trp of the metabolism of tryptophan by the gut microbiota has a dual role in AD: on the one hand, its metabolites (such as IPA) play a neuroprotective role by reducing Aβ deposition and neuroinflammation, while improving cognitive function; on the other hand, metabolic abnormalities (such as excessive Trp levels or an imbalance in the KYN/Trp ratio) may aggravate the pathological process of AD. These effects are mainly mediated by MGBA, involving a variety of pathways, including immunomodulation, neurotransmitter balance and oxidative stress (Li et al., 2022; Sun et al., 2022). Based on existing research, future research on gut microbiota-tryptophan metabolism axis in AD should focus on transforming the understanding of the mechanism of “gut microbiota-metabolites-brain” interaction into clinical intervention strategies. Efforts should be made to combine circulating Trp metabolites with neuroimaging and cognitive scales to establish a multi-dimensional biomarker system for early warning, staging and therapeutic effect evaluation of AD. The ultimate goal is to develop new AD prevention and treatment routes based on individualized intestinal metabolic spectrum.

### 3.2 PD

Parkinson’s disease is a common NDDs in the elderly. Its clinical manifestations include sports and non-motor symptoms. Motor symptoms mainly include static tremor, muscle tonicity, motor retardation and posture instability; non-motor symptoms include sensory disorders, sleep disorders and psychiatric symptoms (Bloem et al., 2021). Research shows that tryptophan metabolism is disturbed in patients with PD. It leads to an increase in the generation of QUIN. This interference exacerbates neuroinflammation through multiple mechanisms, including activating N-methyl-D-aspartic acid receptors (NMDAR), promoting the production of active oxygen (ROS), inducing lipid peroxidation, and increasing the level of nitric oxide synthase (Chang K. H. et al., 2018; Behl et al., 2021). Inflammation is the key link between gut microbiota and PD. Imbalance of Trp metabolites will exacerbate the inflammatory response of the intestine and brain, thus accelerating the progression of symptoms (Fan et al., 2025). In addition, increased levels of QUIN and decreased levels of KYNA in animal models of PD have been shown to exacerbate the damage of dopaminergic neurons in the substantia nigra pars compacta (SNpc), the primary brain region affected in PD (Poeggeler et al., 2007). Mir et al. (2020) proved that indole, a metabolite of gut microbiota, can improve the motor function of mouse models, which may be achieved by stimulating the vagus nerve. In the process of Trp metabolism, the “triad” composed of tryptophan metabolism, AhR, and the gut microbiota plays a crucial role: the tryptophan-Kynurenic pathway and AhR jointly regulate immune and neurological function, and their abnormalities can aggravate neuroinflammation. For example, patients with PD show upregulation of indoleamine 2,3-dioxygenase 1 (IDO-1), which promotes pro-inflammatory reactions and accelerates neurodegeneration (Iwaniak et al., 2024). Furthermore, an imbalance in gut microbiota can disrupt the equilibrium of Trp metabolism, potentially influencing systemic inflammation levels through the regulation of Trp metabolites, such as indole sulfate. Research conducted by Zhou et al. (2023) indicates that the gut microbiota composition in patients with PD undergoes changes, notably the proliferation of indole-producing bacteria, which correlates with elevated plasma levels of Trp metabolites, including indole sulfate. These alterations may serve as early biomarkers or could expedite disease progression via inflammatory mechanisms. Additionally, gut microbiota impacts the development of PD through immune pathways. The AhR, which is extensively expressed in immune cells, interacts with metabolites generated from the three metabolic pathways of Trp, derived from gut microbiota, acting as ligands to facilitate immune regulation. For instance, Westfall et al. (2017) demonstrated that KYNA and QUIN may exert immune effects within the MGBA.

**FIGURE 3:**
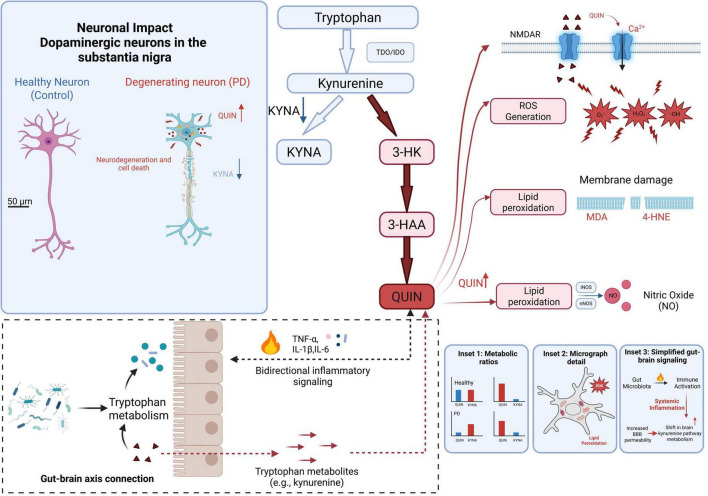
Tryptophan-kynurenine metabolic disorder in Parkinson’s disease (PD) patients.

Future research on the gut microbiota-tryptophan metabolic axis in PD should prioritize elucidating the specific temporal and spatial mechanisms underlying metabolic disorders. This is particularly important for understanding the imbalance between the neurotoxic product QUIN and the neuroprotective product KYNA in the KP, which drives both central and peripheral inflammation, ultimately leading to selective damage to dopaminergic neurons. Furthermore, it is essential to investigate whether metabolites derived from gut microbiota, such as indole sulfate, or circulating Trp metabolites can serve as practical biomarkers for the early diagnosis, subtype identification, and monitoring of disease progression. The potential of these metabolites in predicting non-motor symptoms should also be assessed. Ultimately, this research could pave the way for early intervention and personalized treatment strategies for PD (Figure 3).

### 3.3 Other NDDs

#### 3.3.1 ALS

Amyotrophic Lateral Sclerosis, also referred to as motor neuron disease, is a chronic progressive condition characterized by the gradual degeneration of motor neurons. It is characterized by the degeneration of upper and lower motor neurons, which eventually leads to muscle weakness, muscle atrophy and respiratory failure. Its main pathological characteristics include neuroinflammatory response, mitochondrial dysfunction, oxidative stress, excitotoxicity and abnormal RNA processing. The influence of gut microbiota on ALS is primarily manifested in several ways, including RNA processing disorders, oxidative stress, axonal transport dysfunction, neuroinflammation, endoplasmic reticulum stress, and abnormal immune responses. Among them, the typical manifestations of RNA processing disorders in NDDs include: splicing defects related to TAR DNA-binding protein 43 (TDP-43) and fused in sarcoma (FUS) in ALS/frontotemporal dementia (FTD), splicing abnormalities related to RNA foci in C9ORF72-related ALS/FTD (Rummens and Da Cruz, 2025; Zeng et al., 2025). Dysbiosis of gut microbiota plays a crucial role in the pathogenesis of ALS, as it not only accelerates disease progression but also worsens with the advancement of ALS, thereby establishing a vicious cycle. Gut microbiota dysbiosis promotes neuroinflammation, which, in turn, facilitates the onset of ALS through mechanisms involving reactive oxygen species, activation of the innate immune system, pro-inflammatory cytokines, and infiltration of immune cells. This cascade accelerates the death of motor neurons and releases detrimental factors such as damage-associated molecular patterns and protein aggregates. These factors can further intensify neuroinflammation, thereby perpetuating a cycle of progressive neuroinflammation in ALS (Thonhoff et al., 2018; Chidambaram et al., 2022). The latest animal studies show that changes in the microbial community can affect the severity of the disease, and the beneficial strain *Akkermansia muciniphila* is associated with metabolic changes that may improve the ALS-related phenotype (Feng et al., 2025). Although the existing evidence is still limited, these findings support the possibility that metabolic regulation mediated by intestinal microbiota (including tryptophan-related pathways) may be involved in neuroinflammatory reactions and disease progression in ALS. Future studies need to clarify its causal mechanism and determine whether specific microbial metabolites can be used as therapeutic targets or biomarkers.

#### 3.3.2 HD

Huntington’s disease is a NDDs inherited by autosomal dominance. It is characterized by the production of abnormal huntingtin protein due to the mutation of the huntingtin protein (HTT) gene. This abnormal protein will damage the function of the basal ganglia, and then cause motor, cognitive and mental disorders (Rüb et al., 2016). Neuroinflammation is a key pathophysiological feature of HD. Metabolites produced under conditions of gut microbiota dysbiosis. These factors can then cross a compromised BBB and induce neuroinflammation (Zhang et al., 2018). Keene et al. (2001) in the HD rat model induced by 3-nitropropionic acid (3-NP), it was found that endogenous hydrophilic bile acid tauroursodeoxycholic acid (TUDCA) can reduce cell apoptosis in the HD model, reduce 3-NP-mediated neuron death, and maintain the morphology of striatal mitochondria; However, its exact mechanism is not yet clear. In short, gut microbiota metabolites may be involved in regulating neuroinflammation and mitochondrial function in HD. In this case, the metabolism of Trp by the gut microbiota may affect neuroinflammation, and changes in bile acid metabolism may further affect microbial and host metabolic pathways involved in gut-brain communication. A recent review suggests that the metabolic network disorders associated with Trp and bile acids may jointly contribute to abnormal intestinal function, systemic inflammation and CNS pathology in patients with HD (Gu et al., 2026). However, the relevant evidence is not sufficient at present, and more research is needed to clarify the interaction mechanism of these metabolic pathways and whether these pathways can serve as potential therapeutic targets.

For other NDDs, such as ALS and HD, the study of the gut microbiota-tryptophan metabolic axis is still in the exploration stage. Future research should systematically clarify how gut microbiota disorders specifically drive Trp metabolism and reorganization in these disease models, and clarify the key mechanisms of their metabolites affecting core pathologies such as motor neuron death, abnormal protein aggregation and mitochondrial dysfunction, so as to break the malignancy between microbial dysbiosis and neuroinflammation cycle. At the same time, it is necessary to actively explore the potential therapeutic effect of common intervention strategies for the above diseases. In addition, efforts should be made to identify specific intestinal microorganisms or their metabolic biomarkers for early warning or disease progression monitoring, so as to open up new ways to prevent and treat these diseases that currently lack effective treatments.

### 3.4 Comparison of tryptophan metabolic alterations across NDDs

Although disturbances in Trp metabolism have been reported in multiple neurodegenerative diseases, the direction and magnitude of metabolite changes are not entirely consistent across studies. Overall, a shift toward the KP, often reflected by an increased KYN/Trp ratio, higher levels of metabolites associated with neurotoxic effects, such as QUIN, and/or lower levels of metabolites that may exert neuroprotective effects, such as KYNA or microbiota-derived indole derivatives, appears to be a common feature associated with neuroinflammation and neurodegeneration (Chang K. H. et al., 2018). However, the reported levels of specific metabolites may differ among studies. These discrepancies may be explained by several factors, including differences in study populations (e.g., age, sex, diet, medication use, and comorbidities), disease stage and severity, biological sample type, and analytical approaches used for metabolite detection. In addition, since Trp metabolism is dynamically regulated by both host inflammatory status and gut microbial composition, metabolite profiles may vary substantially across cohorts and disease stages (González-Domínguez et al., 2015).

Across different NDDs, several common features can be identified, including enhanced KP activity, impaired production of neuroprotective indole metabolites, and disruption of immune and barrier homeostasis. At the same time, disease-specific patterns may also exist. In AD, alterations in indole derivatives and serotonin-related signaling may be more closely associated with cognitive decline, synaptic dysfunction, and Aβ/tau pathology (Xie et al., 2024). In PD, KP imbalance and gut barrier dysfunction may be more strongly linked to α-synuclein aggregation, dopaminergic neuronal injury, and motor/non-motor symptoms (Venkatesan et al., 2020). In ALS and HD, the available evidence is still limited, but current studies suggest that gut microbiota-related metabolic dysregulation may contribute to neuroinflammation, mitochondrial dysfunction, and disease progression. These similarities and differences may reflect distinct pathological backgrounds, selective neuronal vulnerability, and microbiota-host interactions in each disorder (Wasser et al., 2020; Chen et al., 2024). The mechanisms of Trp and its metabolites in different NDDs are illustrated in Table 1.

**TABLE 1:** The mechanisms of Trp and its metabolites in different neurodegenerative diseases (NDDs).

Disease	Changes in major tryptophan metabolites	Immunomodulation mechanism	Neuroprotection/toxic effects	Effects on BBB function	Possible explanations for discrepancies	References
AD	Indole derivatives (such as IPA) increased KYN/Trp ratio	Indole can activate AhR and inhibit neuroinflammation; it can reduce Aβ-induced microglial activation.	IPA can reduce Aβ deposition and tau protein phosphorylation; it can improve synaptic plasticity.	Indole derivatives affect the permeability of the BBB through AhR; inflammation will aggravate the barrier damage.	Differences in disease stage (prodromal vs. established AD), dietary Trp intake, gut microbiota composition, plasma vs. CSF sampling, and analytical platforms	Sun et al., 2022; Owe-Larsson et al., 2025; Salminen, 2023; Chyan et al., 1999; Wu et al., 2021
PD	The KP may be shifted toward the neurotoxic branch (QUIN↑, KYNA↓); the indole sulfate is elevated.	IDO-1 is upregulated and promotes inflammation; AhR ligand imbalance exacerbates neuroinflammation.	QUIN promotes α-synuclein aggregation; indole derivatives improve intestinal barrier integrity and delay pathological progression	Increased intestinal permeability will promote the spread of α-synuclein; damage to the BBB will aggravate inflammation.	Variability in motor vs. non-motor phenotypes, medication exposure, gut permeability status, inflammatory burden, and cohort size	Iwaniak et al., 2024; Fan et al., 2025; Jurcau et al., 2023; Zhou et al., 2023; Chang K. H. et al., 2018; Behl et al., 2021; Poeggeler et al., 2007; Mir et al., 2020
ALS	Overall disorder of Trp metabolism; increased inflammation-related metabolites	Microbial dysbiosis promotes systemic and neuroinflammation and activates the TLR/NF-κB pathway.	Oxidative stress and mitochondrial dysfunction can aggravate the death of motor neurons.	Impaired intestinal barrier integrity will cause endotoxins to transfer to the blood and aggravate neuroinflammation.	Small sample size, limited metabolomics data, heterogeneous clinical subtypes, and lack of longitudinal studies	Thonhoff et al., 2018; Chidambaram et al., 2022
HD	The metabolic pathway of Trp has not been fully clarified; it may interact with bile acid metabolism.	Microbial metabolites enter the brain through a disrupted BBB, activate microglia, and promote striatal inflammation.	Bile acids (such as TUDCA) may protect mitochondrial function and reduce neuronal apoptosis.	Damage to the integrity of the BBB will promote peripheral inflammatory mediators to enter the brain.	Limited direct evidence, reliance on animal models, and differences in experimental design and endpoints	Zhang et al., 2018; Keene et al., 2001

## 4 Intervention strategies and therapeutic potential

### 4.1 Nutritional intervention

Specific nutritional interventions may play a protective role against NDDs by regulating Trp metabolism. Pan et al. (2024) confirmed in male APP/PS1 mice that a high Trp diet (0.5% Trp) significantly reduced cognitive disorders and Aβ deposition by upregulating the AhR pathway and inhibiting the NF-κB signaling pathway. Similarly, Yin et al. (2021) confirmed in elderly mice that a high Trp diet (0.4% Trp) can reduce oxidative stress and neuroinflammation, while improving nerve damage. In addition, a high-fiber diet can regulate the composition of gut microbiota, enhance its diversity, promote the proliferation of beneficial bacteria, and increase the production of short-chain fatty acids (SCFA). By enhancing the synthesis and inhibition of the inflammatory pathway of 5-HT, they optimize Trp metabolism and mediate serotonin-dependent insulin sensitivity improvement (Li et al., 2024), indicating that a high-fiber diet may also be a potential AD protection mechanism. Future research must transform nutritional intervention from animal models to clinical verification, focusing on how diet can accurately regulate the Trp metabolism network and its synergistic mechanism. This should include develop personalized nutritional strategies and therapeutic biomarkers based on individual microbiome and metabolic spectrum to determine their practical application value in the prevention and treatment of NDDs.

However, several important limitations should be acknowledged. First, dose-dependent effects and potential toxicity remain a major concern. Given the complexity of Trp metabolism, excessive supplementation may shift metabolic flux toward the production of neurotoxic metabolites, such as QUIN, rather than beneficial indole derivatives, and the optimal therapeutic window has yet to be defined. Second, substantial inter-individual variability is likely to influence treatment response. Baseline gut microbiota composition, host genetic polymorphisms (e.g., in IDO or AhR-related genes), and disease stage may all shape the metabolic and clinical effects of dietary interventions. Therefore, strategies that show efficacy in relatively homogeneous animal models may not be readily translatable to heterogeneous human populations. Third, clinical evidence remains limited. To date, no large-scale randomized controlled trials have specifically evaluated the effects of high-tryptophan or high-fiber diets in patients with NDDs. In addition, long-term dietary intervention studies are inherently challenging because blinding, standardization, and adherence are difficult to maintain. Finally, potential drug-diet interactions should not be overlooked. High-tryptophan diets may interact with medications commonly used in patients with NDDs, such as selective serotonin reuptake inhibitors or monoamine oxidase inhibitors, thereby raising safety concerns.

Future studies should focus on developing personalized nutritional strategies guided by baseline metabolomic and microbiome profiling. At the same time, rigorous dose-finding studies are needed to determine the safety, tolerability, and efficacy of tryptophan- or fiber-based dietary interventions in different NDDs settings.

### 4.2 Microbiome-targeted therapies

**FIGURE 4:**
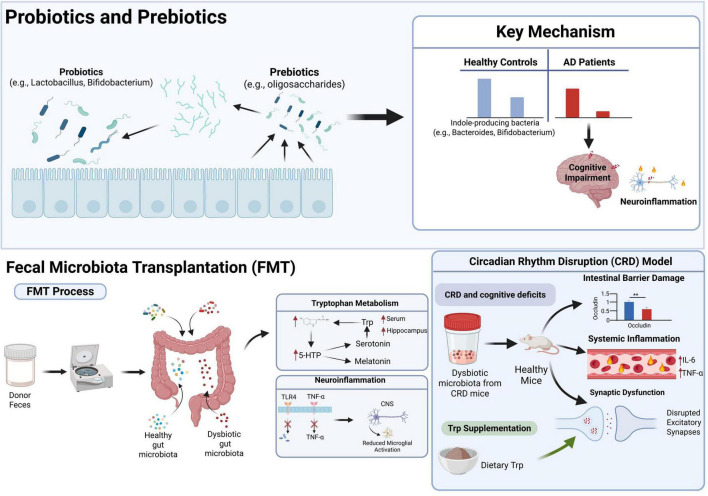
Trp metabolite-targeted therapy in modulating neurodegenerative diseases.

The development and progression of NDDs are closely linked to an imbalance in gut microbiota. Regulating gut microecology through targeted Trp metabolites derived from microbiota therapy has emerged as a promising intervention strategy (Figure 4).

#### 4.2.1 Probiotics and prebiotics

Probiotics (such as *Lactobacillus* and *Bifidobacterium*) are active beneficial microorganisms that can be directly supplemented, while prebiotics (such as dietary fiber, oligosaccharides) selectively promote the growth of probiotics (Sanders et al., 2019). Clinical observations show that compared with healthy individuals, the level of some indole-producing bacteria (such as *Mycobacterium* and *Bifidobacterium*) in the intestines of patients with AD is reduced (Guo et al., 2021). This indicates that some intestinal bacteria can increase the production of indole, thus reducing neuroinflammation and improving cognitive disorders.

The latest research shows that probiotics have therapeutic potential. In the 3xTg-AD mouse model, supplementing Lactobacillus plantarum KY1032 and Lactobacillus curvatus HY7601 can improve memory, reduce the number of astroglial cells and microglial activation, and better protect the hippocampal neurons, indicating that probiotics regulate the imbalance of gut microbiota, which can delay early NDDs (Medeiros et al., 2024). In the PD study, a 12-week randomized double-blind placebo-controlled trial found that the combined supplementation of BF839+ earthworm protein could significantly improve the overall score of PD, while reducing depression, anxiety and constipation without serious side effects (Zeng et al., 2024).

However, there are still many key issues facing. The effect of probiotics and prebiotics largely depends on the type of strain, formula, dosage, duration of use and the patient’s own gut microbiota. Future research needs to focus on clarifying the mechanism of action of different strains, biomarkers that can be used to judge the efficacy, and standardized clinical evaluation indicators.

#### 4.2.2 FMT

Fecal microbiota transplantation is to transplant the functional microbial community in the feces from a healthy donor into the patient’s gastrointestinal tract to rebuild the new gut microbiota, thus treating intestinal and extraintestinal diseases. Xue et al. (2020) Preliminary research on the treatment of PD by FMT shows that FMT can reverse abnormal Trp metabolism and effectively relieve motor and non-motor symptoms. In addition, FMT may delay the progression of the disease by inhibiting neuroinflammation and reducing the TLR4/TNF-α signaling pathway (Sun et al., 2018). Animal studies by Song et al. (2025) show that circadian rhythm disorder (CRD) can lead to cognitive disorders. The gut microbiota of CRD mice is transferred to normal mice through FMT, which can reproduce cognitive defects, intestinal barrier damage (decreased expression of tight junction proteins), systemic inflammation (elevated serum IL-6/TNF-α levels) and pathological changes of CNS. Supplementing dietary Trp (0.5%) can reverse the pathological changes induced by CRD: Restore serum/hippocampal Trp and 5-hydroxytryptophan (5-HTP) levels and improve intestinal barrier function, Inhibit neuroinflammation and glial cell activation, restore synaptic protein expression and excitative synaptic transmission, and ultimately improve cognitive defects. Therefore, FMT may become an auxiliary treatment for NDDs. Targeted by the microbiome Treatment (For example, probiotics/prebiotics, fecal microbiota transplantation) shows the potential to improve NDDs by reshaping intestinal microbiota and Trp metabolism. Future research needs to be rigorously designed. Clinical trials to verify its long-term efficacy and safety; at the same time, it is necessary to clarify the accuracy of regulating intestinal metabolism and immune networks. Mechanism, and Establish a personalized intervention plan based on the characteristics of individual microbiome.

Nevertheless, despite this promise, the clinical translation of microbiome-targeted therapies still faces substantial limitations and challenges. First, the composition and function of the gut microbiota vary considerably among individuals, and such heterogeneity may lead to highly variable therapeutic responses. Second, the long-term safety and durability of these interventions remain unclear. Third, the mechanisms by which microbiome-based therapies regulate Trp metabolism, host immunity, and neuroinflammation have not yet been fully clarified, making it difficult to identify the most effective microbial strains, donor profiles, or treatment regimens. In addition, standardization remains a major obstacle, including differences in donor selection, preparation protocols, dosage, route of administration, treatment frequency, and outcome evaluation. These issues are particularly important in NDDs, where disease progression is slow, patient populations are heterogeneous, and long-term follow-up is required. Therefore, future studies should focus on well-designed clinical trials to evaluate efficacy, safety, and durability, while also clarifying the mechanistic basis of MGBA interactions and developing personalized intervention strategies based on individual microbiome and metabolic profiles.

### 4.3 Metabolite-derived therapeutics

Ischemia-reperfusion (I/R) injury refers to the damage sustained by brain tissue following a temporary interruption of blood flow, which leads to inflammation, oxidative stress, and neuronal death. Natural compounds, such as dihydroflavonoids, have been shown to mitigate I/R-induced neuronal damage by modulating the gut microbiota-tryptophan axis, thereby alleviating both intestinal and systemic inflammation (Peng et al., 2025). Furthermore, as an AhR ligand, IPA can inhibit neuroinflammation, promote nerve regeneration, delay the onset of NDDs and cognitive disorders, and improve disease progression (Owe-Larsson et al., 2025). Recent studies have shown that IPA can inhibit the RAGE-JAK2-STAT3 pathway, reduce neuronal apoptosis and oxidative damage, suppress microglial activation, promote neurogenesis, preserve the integrity of both the BBB and the intestinal barrier, and remodel the gut microbiota, thereby improving memory impairment in lipopolysaccharide (LPS)-induced cognitively impaired mice. At the same time, IPA has also been identified as a key mediator linking gut microbiota remodeling to cognitive improvement and reduced amyloid pathology in an AD mouse model (Hao et al., 2025; Li L. et al., 2025). These findings suggest that metabolite-based therapies may serve not only as downstream markers of microbiota function, but also as active mediators with therapeutic potential.

Despite this potential, similar to other microbiome-based interventions, the clinical translation of metabolite-derived therapeutics still faces several challenges. Their effects may be dose dependent and context specific, and inappropriate supplementation could potentially shift Trp metabolism toward neurotoxic branches. In addition, individual differences in microbiota composition, host metabolism, and disease stage may influence treatment response. Most importantly, current evidence is still largely based on preclinical studies, and robust clinical trials are still lacking to determine the safety, efficacy, and optimal use of IPA and other related metabolites in patients with NDDs. Therefore, further translational studies are needed before these metabolites can be considered for clinical application.

## 5 Conclusion and future directions

Overall, current evidence highlights the gut microbiota-tryptophan-brain axis as a promising framework for understanding the pathogenesis of NDDs. A major insight is that Trp metabolism may provide both mechanistic clues and clinically relevant targets, particularly through AhR signaling, barrier integrity, and immune regulation. These findings also underscore the translational potential of interventions targeting this axis, including dietary modulation, microbiome-based strategies, and metabolite-derived therapies such as FMT and IPA-related approaches.

Future research should be prioritized: (1) Integrate polyomomics (metabolomics, microbiology, transcriptomics) to identify disease-specific tryptophan metabolites characteristics and their upstream microbial drivers; (2) use single-cell sequencing and spatial transcriptomics to clarify tryptophan metabolites and pathological proteins (Aβ, Molecular interaction between α-synuclein); (3) Carry out large-scale prospective clinical trials to verify the application of metabolite-based biomarkers (such as IPA, KYN/KYNA ratio) in early diagnosis and treatment monitoring; (4) Developing precise interventions based on individual metabolic profiles, such as the use of indole probiotics for AD and kynurenine 3-monooxygenase inhibitors for PD. Through these initiatives, it is anticipated that the gut microbiota-tryptophan-brain axis will emerge as a pivotal target for the prevention and treatment of NDDs.
